# Effect of ezetimibe–statin combination therapy vs. statin monotherapy on coronary atheroma phenotype and lumen stenosis in patients with coronary artery disease: a meta-analysis and trial sequential analysis

**DOI:** 10.3389/fphar.2024.1343582

**Published:** 2024-05-13

**Authors:** Yun-Jing Zhang, Min Xu, Ji-Qiang Duan, De-Jin Wang, Shi-Liang Han

**Affiliations:** ^1^ Department of Nephrology, Zibo Central Hospital, Zibo, Shandong, China; ^2^ Department of Orthopedics, Zibo Central Hospital, Zibo, Shandong, China; ^3^ Department of Cardiology, Zibo Central Hospital, Zibo, Shandong, China

**Keywords:** ezetimibe, statin, coronary plaques, atheroma plaque, intravascular ultrasound, optical coherence tomography

## Abstract

**Background:**

Evidence indicates that the addition of ezetimibe to statin therapy reduces cardiovascular events. However, the impact of ezetimibe–statin combination therapy on coronary plaque regression, plaque stabilization, and diameter stenosis remains a matter of controversy.

**Methods:**

We performed electronic searches in PubMed, Web of Knowledge, and the Cochrane Central Register of Controlled Trials to identify eligible trials assessing the effects of ezetimibe–statin combination therapy *versus* statin monotherapy reporting at least one outcome among total atheroma volume (TAV), minimum fibrous cap thickness (FCT), lumen volume (LV), and lumen area (LA) derived from intravascular imaging modalities of intravascular ultrasound (IVUS) and optical coherence tomography (OCT). We used the random-effects model and performed trial sequential analysis (TSA) during this meta-analysis.

**Results:**

Eleven articles with a total of 926 individuals (460 in the dual-lipid-lowering therapy group and 466 in the statin monotherapy group) were included in the final meta-analysis. Compared to statin monotherapy, ezetimibe–statin combination therapy was associated with significantly decreased TAV [WMD = −3.17, 95% CI (−5.42 to −0.92), and *p* = 0.006], with no effect on the LV of the coronary artery [WMD = −0.52, 95% CI (−2.24 to 1.21), and *p* = 0.56], the LA of the coronary artery [WMD = 0.16, 95% CI (−0.10–0.42), and *p* = 0.22], or minimum FCT thickness [WMD = 19.11, 95%CI (−12.76–50.97)].

**Conclusion:**

In patients with coronary artery disease, ezetimibe–statin combination therapy resulted in a significant regression in TAV compared to statin monotherapy, whereas no overall improvements of minimum FCT or lumenal stenosis were observed.

## Introduction

The significance of lipid-lowering therapy in coronary artery disease (CAD) has been established through numerous clinical trials. Statins, which inhibit cholesterol synthesis and increase low-density lipoprotein cholesterol (LDL-C) clearance, are recommended as a first-line agent for patients with cardiovascular (CV) disease. The Cholesterol Treatment Trialist (CTT) meta-analysis showed a 20% reduction in the 5-year incidence of adverse CV events per 1 mmol/L decrease in LDL-C concentration during statin therapy (Baigent et al., 2005; Armitage, 2007). However, despite the efficacy of intensive statin therapy associated with an increased incidence of side effects (Armitage, 2007), atherosclerosis continues to progress in up to one-third of patients, and patients remain exposed to high “residual risk” of future acute CV events (Bayturan et al., 2010). In such cases, it is necessary to combine statin with other kind of lipid-lowering drugs (i.e., dual-lipid lowering therapy).

Ezetimibe is a cholesterol absorption inhibitor that can lower the plasma cholesterol level by reducing cholesterol absorption from the small intestine. In conjunction with statin therapy, it mitigates the enhanced lipid absorption that occurs during the inhibition of cholesterol synthesis (Savarese et al., 2015). Adding ezetimibe to statin therapy has been shown to further reduce plasma LDL-C by 15%–20% (Bohula et al., 2015). Compared with the combination therapy of simvastatin with placebo, daily administration of simvastatin plus ezetimibe led to a significantly lower incidence of the primary combined CV endpoint (CV death, myocardial infarction, re-hospitalization for unstable angina, coronary revascularization, or stroke) (Cannon and Circulation, 2014). However, the underlying mechanism of how adding ezetimibe to statin therapy results in the modification of coronary plaque remains controversial.

The PROSPECT study showed that lesion-related risk factors for major adverse CV events were characterized by thin-cap fibroatheromas, a large plaque burden, a small luminal area, or some combination of these characteristics (Stone et al., 2011). Over the past decade, there have been significant advancements in identifying coronary plaque characteristics by intravascular imaging, including intravascular ultrasound (IVUS) and optical coherence tomography (OCT) (Koskinas et al., 2016). IVUS is suitable for assessing plaque volume, but it lacks the spatial resolution required for precise assessment of fibrous cap thickness (FCT). In contrast, OCT excels in evaluating small changes in FCT, yet its limited tissue penetration makes it unsuitable for volumetric plaque assessment (Kim et al., 2018). Thus, both IVUS and OCT are needed for systematically and comprehensively evaluating coronary plaque characteristics. Despite the strengths of these imaging modalities, as of yet, there is no evidence-based systemic review demonstrating whether adding ezetimibe to statin therapy provides incremental benefits in coronary plaque modification compared to statin monotherapy through meta-analysis on the outcomes derived from both intravascular IVUS and OCT.

Thus, the first aim of this study is to evaluate the effect of ezetimibe–statin combination therapy, compared with statin monotherapy, on the progression/regression of coronary atherosclerotic plaque and lumen volume (LV) measured by IVUS. The second aim is to clarify whether ezetimibe–statin combination therapy is associated with the stabilization of coronary plaque evaluated by FCT through OCT examination. The third aim is to further verify the outcome of the LV derived from IVUS by evaluating the minimum lumen area (LA) through OCT examination.

## Materials and methods

The present review was conducted strictly according to “Handbook for Systematic Reviews of Interventions Version 5.1.0” (Higgins and Green, 2013) and reported its findings following the Preferred Reporting Items for Systematic Reviews and Meta-Analyses (PRISMA) guidelines for reporting systematic reviews (Liberati et al., 2009).

### Literature search

We performed a comprehensive search in the Cochrane Central Register of Controlled Trials (CENTRAL), Web of Knowledge, and PubMed databases (up to November 2023). To ensure a thorough and systematic examination of all relevant studies, both medical subject heading terms (MeSH) and keywords were used, including ezetimibe, optical coherence tomography (OCT), intravascular ultrasound (IVUS), coronary plaque, and atheroma plaque. The search outcome was restricted to studies published in English. Additionally, a manual search was performed by cross-checking the reference list of the selected articles. We updated the search strategy before our manuscript submission to ensure a comprehensive investigation. Given that this is a meta-analysis based on data collected from published papers, ethics approval was deemed unnecessary.

### Inclusion and exclusion criteria

All search items were evaluated for eligibility by two reviewers (YJ Zhang and SL Han). Consensus was reached by negotiation.

### Inclusion criteria

To be included in the final review, the following criteria should be met:

1. Type of studies: Randomized or non-randomized controlled trials, prospective or retrospective cross-sectional studies;
2. Participants: Patients with all types of coronary atherosclerotic artery disease, regardless of disease duration and severity, gender, age, prior cardiovascular medications, or region;
3. Comparation: Ezetimibe–statin combination therapy *versus* statin monotherapy;
4. Outcome: (1) IVUS used as a modality for measuring absolute changes from baseline in total atheroma volume (TAV) and lumen volume (LV); (2) OCT used as a modality for measuring absolute changes from baseline in minimum FCT and minimum lumen area (LA).

### Exclusion criteria

Publications of case reports, letters to the editor, meeting abstracts, and correspondence that did not report explicit data were excluded. Articles unrelated to the aim of our topic or published repeatedly were also excluded. In cases where two or more articles reported on overlapping patients, only the article with the largest sample size was included.

### Assessment of risk of bias

The Cochrane recommended “risk of bias table” was used for quality assessment by two investigators (YJ Zhang and SL Han) (Higgins et al., 2011). Each eligible study underwent grading for risk of bias (low, high, and unclear) in six domains: random sequence generation, allocation concealment, blinding of patients and personnel, blinding of outcome assessment, incomplete outcome data, and selective reporting risk. Disagreements were resolved through consultation with a third reviewer (DJ Wang).

### Data extraction

Initially, two investigators (YJ Zhang and SL Han) independently extracted data using a structured study recording form. The extracted study design characteristics included the first author’s name, year of publication, study design, sample volume, baseline demographic characteristics of patients, drug dosage and administration method, prior statin history, duration of follow-up, and main outcomes of interest. A double-check procedure was performed to ensure data accuracy. Finally, a manager (Min Xu) entered the extracted data into a spreadsheet.

### Definition of primary and secondary outcomes

The primary outcome was the change in TAV and LV between baseline and follow-up through IVUS examination. The second outcome focused on the change in minimum FCT and minimum LA through OCT examination. Both IVUS and OCT were performed in non-culprit target lesions of the coronary artery.

For IVUS, the cross-sectional area (CSA) of the external elastic membrane (EEM) and intravascular lumen volume (LV) were measured according to the standards of the American College of Cardiology (Mintz et al., 2001). TAV was defined as the EEM volume minus lumen volume. Based on reproducible landmarks, the same segments were identified and analyzed in the baseline and follow-up IVUS examinations.

For OCT, FCT and LA were evaluated at 1-mm longitudinal intervals to determine the minimum FCT site and the minimum LA site. FCT was defined as the distance from the intimal-lumen border to the lumen edge of the lipid pool characterized by a rapid rise in attenuation. The frame with the minimum FCT and minimum LA at baseline was matched with the corresponding frame at follow-up.

### Statistical analysis

We calculated weighted mean differences (WMDs) for continuous outcomes along with the 95% confidence intervals (CIs). In the absence of reported standard errors, we calculated the standard error of mean difference according to the methods described in the Cochrane Handbook. Prior to data analysis, heterogeneity was assessed by the Cochran Q test and quantified by the I^2^ test. A fixed-effect model was used when the effects were assumed to be homogenous (*p* > 0.05 or I^2^<50%). However, recognizing substantial variations in treatment efficiency related to follow-up duration, prior medication history (e.g., prior statin use or non-use), eligible trial designs, and diverse clinical settings (e.g., stable angina pectoris, acute coronary syndrome) across studies, we assumed the presence of heterogeneity. Consequently, a random-effects model (inverse-variance model) was utilized in all subsequent analyses, the outcome of which is more conservative as it considers differences both within and among studies when calculating the error term used in the analysis. Funnel plots were employed for detecting publication bias, in which the effect sizes (e.g., WMD) are plotted on the horizontal axis, and the associated variances (e.g., the standard error of the intervention effect) are plotted on the vertical axis. Asymmetry around the pooled WMD indicated potential bias.

All statistical analyses were conducted with Review Manager 5.1.0 (Cochrane Collaboration, Oxford, UK). Results were regarded as statistically significant if *p* < 0.05. Finally, trial sequential analysis (TSA) was performed on the associated results using the TSA 0.9.5.10 Beta software, with parameters set as follows: two-side boundary type, Type 1 Error α = 5%, Type 2 Error β = 20%, and statistical power 1-β = 80%. The information axis was based on sample size.

## Results

### Trial flow

**FIGURE 1:**
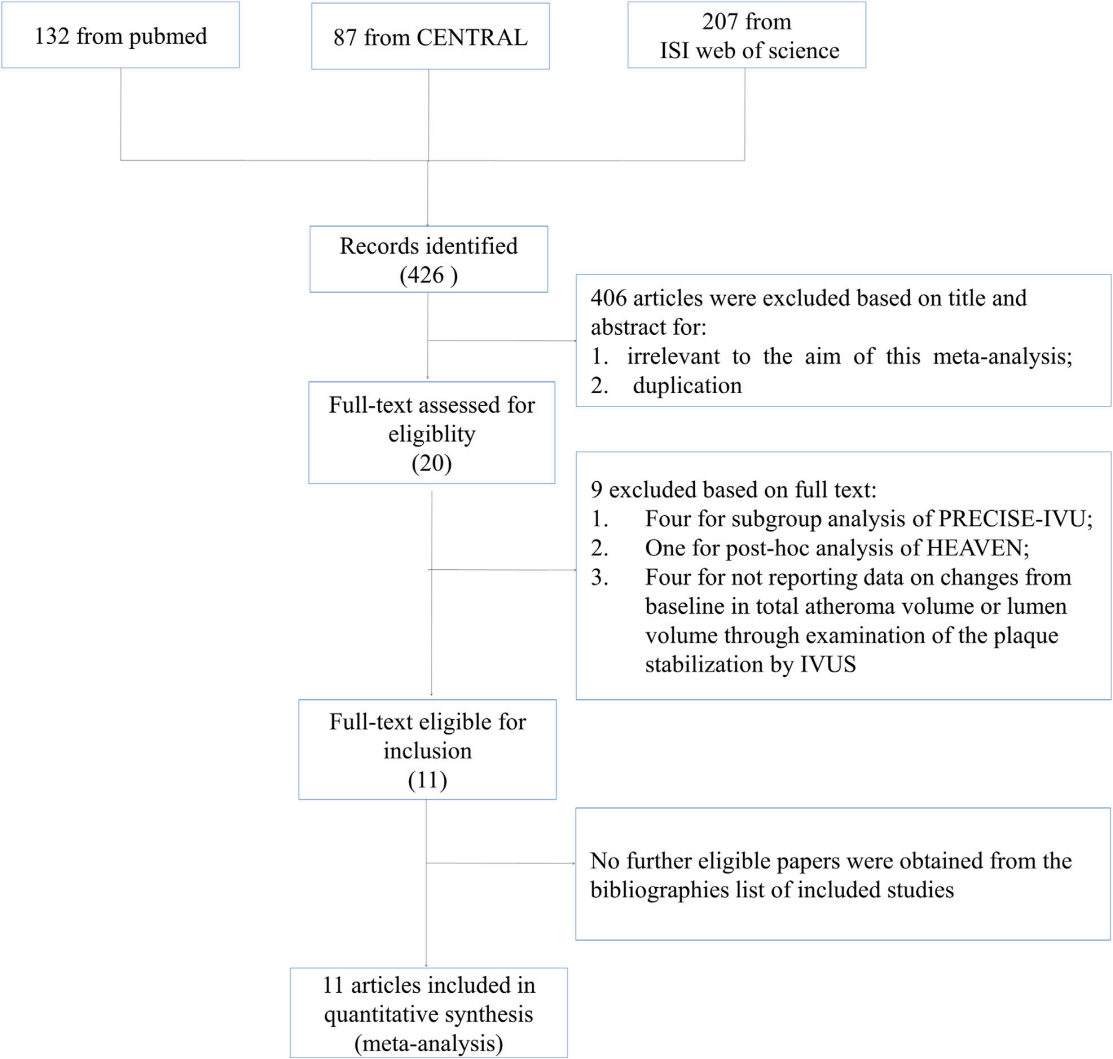
Study selection flow diagram.

A total of 426 citations were generated from the search strategy (132 from PubMed, 207 from ISI Web of Science, and 87 from CENTRAL). After reviewing the titles and abstracts, 406 articles were excluded. Full articles were obtained for the remaining 20 publications. According to the inclusion criteria, nine articles were excluded, with five articles excluded for conducting subgroup or *post hoc* analysis of included studies (Tsujita et al., 2016a; Tsujita et al., 2016b; Kovarnik et al., 2017; Fujisue et al., 2018; Fujisue et al., 2021), and four excluded for comparing the impact of ezetimibe–statin combination treatment *versus* statin monotherapy through IVUS examination without reporting data on TAV or LV (Wang et al., 2016; Ueda et al., 2017; Zhao et al., 2021; Nakano et al., 2023). No further eligible papers were obtained from the bibliographies of the included studies. The study selection process and reasons for exclusions are explicitly described in Figure 1.

### Study characteristics and quality

**FIGURE 2:**
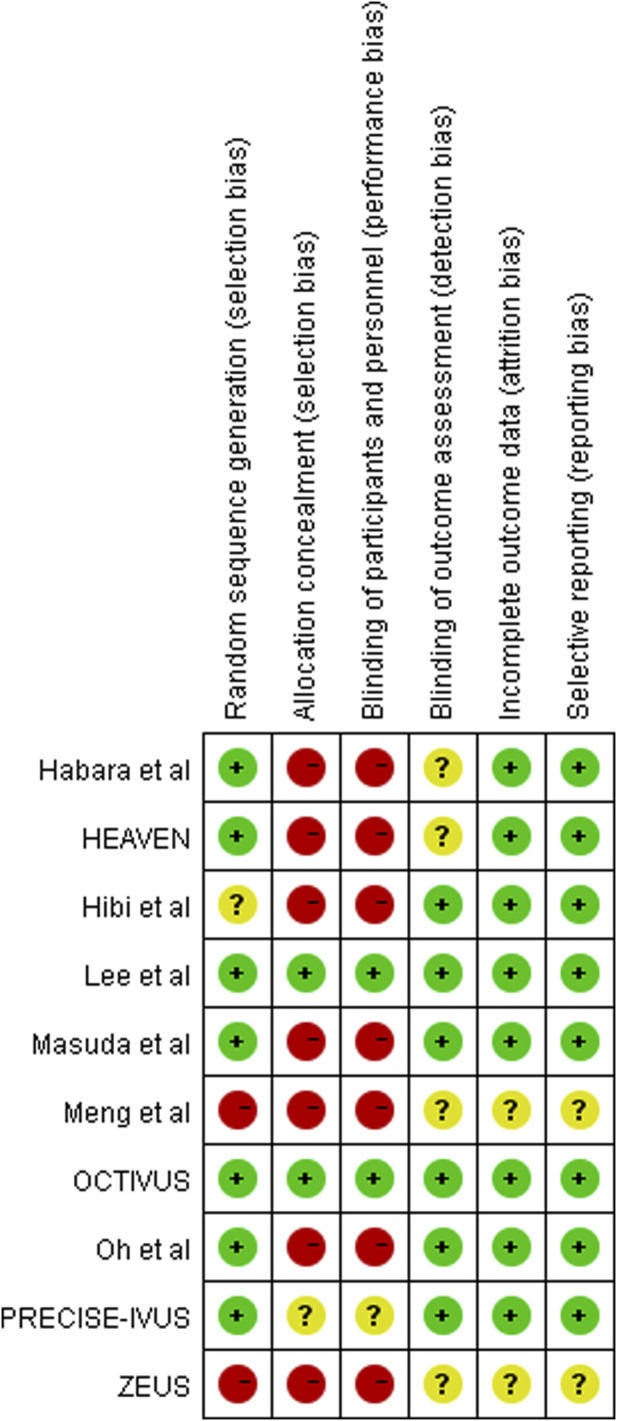
Risk of bias assessment of included studies.

In total, 926 individuals (460 in the ezetimibe–statin combination therapy group and 466 in the statin monotherapy group) were included in the 11 eligible articles (Kovarnik et al., 2012; Habara et al., 2014; Nakajima et al., 2014; Masuda et al., 2015; Tsujita et al., 2015; Lee et al., 2016; Hougaard et al., 2017; Hibi et al., 2018; Hougaard et al., 2020; Meng et al., 2020; Oh et al., 2021), comprising nine RCTs (Habara et al., 2014; Masuda et al., 2015; Tsujita et al., 2015; Lee et al., 2016; Hougaard et al., 2017; Hibi et al., 2018; Hougaard et al., 2020; Oh et al., 2021), one non-randomized trial (Nakajima et al., 2014), and one retrospective trial (Meng et al., 2020). Eight studies involving 723 participants (358 in the dual-lipid-lowering therapy and 365 in the statin monotherapy group) reported outcomes on IVUS (Nakajima et al., 2014; Masuda et al., 2015; Tsujita et al., 2015; Lee et al., 2016; Hougaard et al., 2017; Hibi et al., 2018; Oh et al., 2021), and three studies with a total of 190 participants (97 in the dual-lipid-lowering therapy and 93 in statin monotherapy group) reported outcomes on OCT (Habara et al., 2014; Hougaard et al., 2020; Meng et al., 2020). All eligible studies focused on CAD, with four studies in stable angina pectoris (SAP) (Habara et al., 2014; Masuda et al., 2015; Oh et al., 2021), three in acute coronary syndrome (ACS) (Nakajima et al., 2014; Lee et al., 2016; Hibi et al., 2018), two in CAD (Tsujita et al., 2015; Meng et al., 2020), and two in ST-segment elevation myocardial infarction (STEMI) (Hougaard et al., 2017; Hougaard et al., 2020). In particular, four trials were conducted in patients without a history of statin use before enrollment (Lee et al., 2016; Hougaard et al., 2017; Hibi et al., 2018; Hougaard et al., 2020). Six trials compared ezetimibe plus atorvastatin combination therapy *versus* atorvastatin monotherapy (Nakajima et al., 2014; Tsujita et al., 2015; Hougaard et al., 2017; Hougaard et al., 2020; Oh et al., 2021), one trial compared ezetimibe plus rosuvastatin combination therapy *versus* rosuvastatin monotherapy (Masuda et al., 2015), one trial compared ezetimibe plus simvastatin combination therapy *versus* simvastatin monotherapy (Lee et al., 2016), one trial compared ezetimibe plus pitavastatin combination therapy *versus* pitavastatin monotherapy (Hibi et al., 2018), one trial compared ezetimibe plus fluvastatin combination therapy *versus* fluvastatin monotherapy (Habara et al., 2014), and one trial compared ezetimibe plus rosuvastatin or atorvastatin combination therapy *versus* rosuvastatin or atorvastatin monotherapy (Meng et al., 2020). The mean follow-up duration ranged from 3 to 12 months. Characteristics of eligible studies are shown in Table 1, and the results of the risk of bias assessment are summarized in Figure 2.

**TABLE 1:** Characteristics of studies included in this meta-analysis.

Study	Design	Participants	Dual-lipid-lowering therapy				Statin monotherapy				Duration (months)	Outcome
			N (M/F)	Age	Treatment	Statin naïve	N (M/F)	Age	Treatment	Statin naïve		
Intravascular ultrasound (IVUS)												
Kovarnik et al. (2012)	Single blinded RCT	SAP, non-culprit target lesion with 20%–50% stenosis	42 (9/33)	63.5 ± 9.3	Atorvastatin 80 mg/day + ezetimibe 10 mg/day	43.9%	47 (14/31)	65.1 ± 10.6	Standard statin therapy	34%	12	TAV, LV
Nakajima et al. (2014)	Open-label non-RCT	ACS, emergency PCI in a culprit lesion, non-culprit target lesion ≤25% stenosis	50 (40/10)	63.7 ± 12.6	Atorvastatin 20 mg/day + ezetimibe 10 mg/day	NR	45 (38/7)	60.7 ± 10.5	Atorvastatin 20 mg/day	NR	6	TAV, LV
Masuda et al. (2015)	Open-label RCT	SAP, LDL-C ≥100 mg/dL, non-culprit target lesion ≤50% luminal diameter narrowing	21 (19/2)	64.0 ± 7.9	Rosuvastatin 5 mg/day + ezetimibe 10 mg/day	57.1%	19 (16/3)	70.2 ± 7.6	Rosuvastatin 5 mg/day	63.2%	6	TAV, LV
Tsujita et al. (2015)	Single blinded RCT	ACS and SAP who underwent CAG or PCI under IVUS guidance, LDL-C ≥100 mg/d	100 (80/20)	66 ± 10	Atorvastatin + ezetimibe 10 mg/day	54%	102 (78/24)	67 ± 10	Atorvastatin	51%	9 to 12 (10.1 ± 1.8 vs 9.7 ± 1.7)	TAV, LV
Lee et al. (2016)	Open-label RCT	ACS who had emergency PCI in a culprit lesion, non-culprit target lesion ≤50% luminal diameter narrowing	34 (27/7)	60.9 ± 10.9	Simvastatin 40 mg/day + ezetimibe 10 mg/day	Statin naïve	36 (27/9)	59.3 ± 10.7	Pravastatin 20 mg/day	Statin naïve	3	TAV, LV
Hougaard et al. (2017)	Double blinded RCT	STEMI, non-culprit target lesion with >20% and <50% luminal diameter narrowing	43 (39/4)	55.3 ± 11	Atorvastatin 80 mg/day + ezetimibe 10 mg/day	Statin naïve	44 (36/8)	57.2 ± 9.1	Atorvastatin 80 mg/day	Statin naïve	12 (353 ± 14 vs 356 ± 13 days)	TAV, LV
Hibi et al. (2018)	Open-label RCT	ACS, mild–moderate stenosis in the non-culprit vessel	50 (41/9)	63 ± 10	Pitavastatin 2 mg/day + ezetimibe 10 mg/day	Statin naïve	53 (41/12)	63 ± 12	Pitavastatin 2 mg/day	Statin naïve	8 to 12 (10.0 ± 1.9)	TAV, LV
Oh et al. (2021)	Open-label RCT	SAP, non-culprit target lesion 30%–60% luminal diameter, LDL-C >70 mg/dL	18 (13/5)	56.3 ± 7.1	Atorvastatin 10 mg/day + ezetimibe 10 mg/day	56%	19 (15/4)	56.7 ± 8.4	Atorvastatin 40 mg/day	53%	12 ± 1	TAV, LV
Optical Coherence Tomographic (OCT)												
Habara et al. (2014)	Open-label RCT	SAP with non-culprit target lesion 25%–75% luminal diameter	32 (21/11)	69.8 ± 7.8	Fluvastatin 30 mg/day + ezetimibe 10 mg/day	59%	31 (26/5)	68.8 ± 7.8	Fluvastatin 30 mg/day	49%	9 (285.8 ± 54.4 vs 291.3 ± 27.1day)	FCT, LA
Meng et al. (2020)	Retrospective study	CAD in which the target lesion had a minimum FCT<65 ㎛and lipid core>90°	27 (19/8)	63.56 ± 7.50	Rosuvastatin 5–10 mg or atovastatin 10–20 mg + ezetimibe 10 mg/day	NR	26 (20/6)	64.31 ± 8.53	Rosuvastatin 15–20 mg or atorvastatin 30–40 mg/day	NR	12	FCT, LA
Hougaard et al. (2020)	Double blinded RCT	STEMI with non-culprit target lesion 20%–50% luminal diameter	43 (39/4)	55.3 ± 11	Atorvastatin 80 mg/day + ezetimibe 10 mg/day	Statin naïve	44 (36/8)	57.2 ± 9.1	Atorvastatin 80 mg/day	Statin naïve	12 (354.2 ± 13.3 days)	FCT

RCT, randomized controlled trial; CAD, coronary artery disease; SAP, stable angina pectoris; ACS, acute coronary syndrome; STEMI, ST-segment elevation myocardial infarction; PCI, percutaneous coronary intervention; CAG, coronary angiography; TAV, total atheroma volume; LV, lumen volume; FCT, fibrous cap thickness; LA, lumen area.

### Intravascular ultrasound (IVUS) findings

**FIGURE 3:**
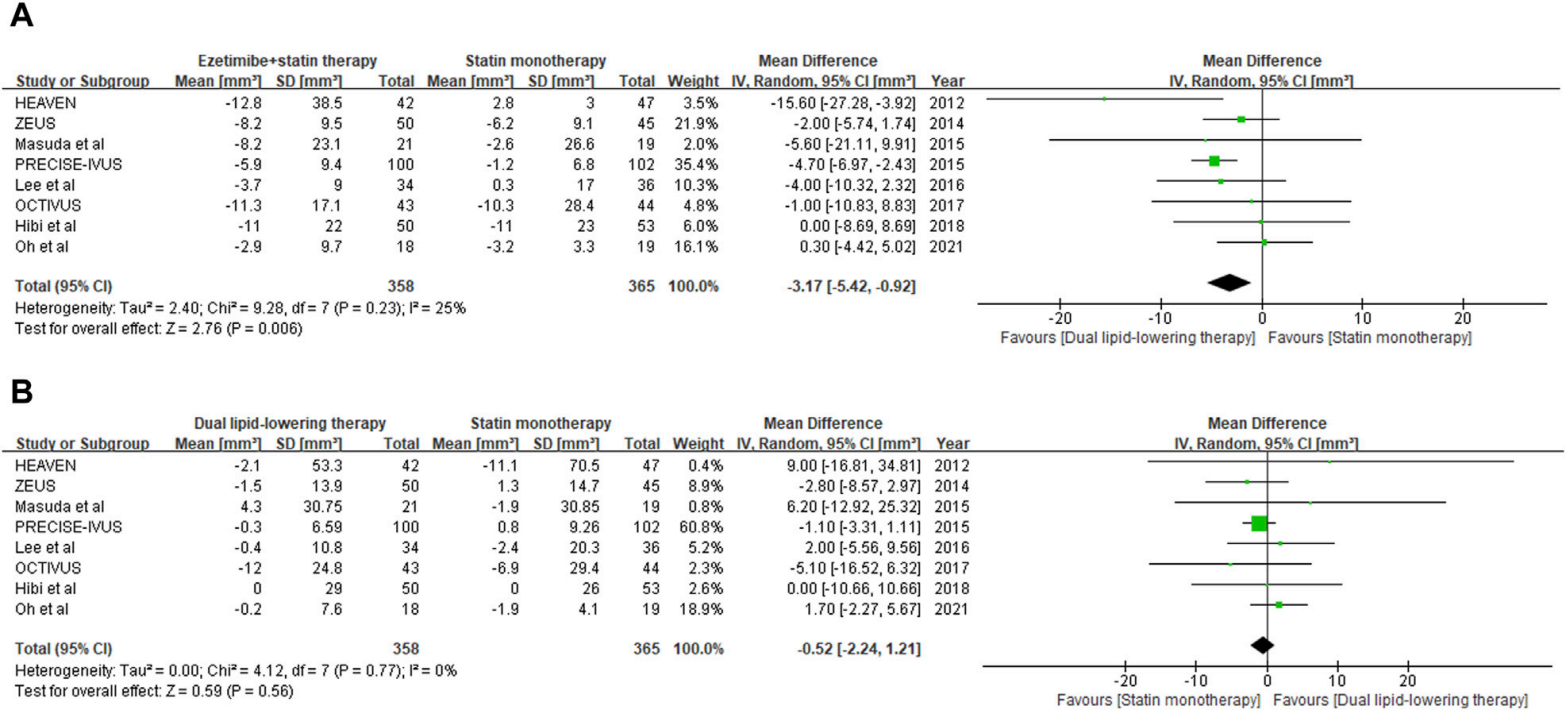
Forest plot of comparison showing change from baseline in total atheroma volume **(A)** and lumen volume **(B)** assessed by IVUS.

Regression or progression of the target plaque lesion was evaluated by TAV through examination with IVUS. Eight studies with 724 individuals (358 in the lipid-lowering therapy group and 365 in the statin monotherapy group) were available at the last follow-up, which included the change from baseline in TAV as an outcome (Nakajima et al., 2014; Masuda et al., 2015; Tsujita et al., 2015; Lee et al., 2016; Hougaard et al., 2017; Kovarnik et al., 2017; Hibi et al., 2018; Oh et al., 2021). With the pooled WMD of −3.17 (95% CI: 5.42 to −0.92), this study demonstrated a significant decrease in TAV (*p* = 0.006) with ezetimibe–statin combination therapy when compared with statin monotherapy, as shown in Figure 3A.

Lumen stenosis of the target coronary artery plaque lesion was further evaluated by LV through examination with IVUS. Eight studies with 724 individuals (358 in the lipid-lowering therapy group and 365 in the statin monotherapy group) were available at the last follow-up, which included the change from baseline in LV as an outcome (Nakajima et al., 2014; Masuda et al., 2015; Tsujita et al., 2015; Lee et al., 2016; Hougaard et al., 2017; Kovarnik et al., 2017; Hibi et al., 2018; Oh et al., 2021). With the pooled WMD of −0.52 (95%CI: 2.24 to 1.21), it was indicated that ezetimibe–statin combination therapy and statin monotherapy had a similar effect on lumenal stenosis of coronary artery (*p* = 0.56), and the outcome is summarized in Figure 3B.

### Optical coherence tomography (OCT) findings

**FIGURE 4:**
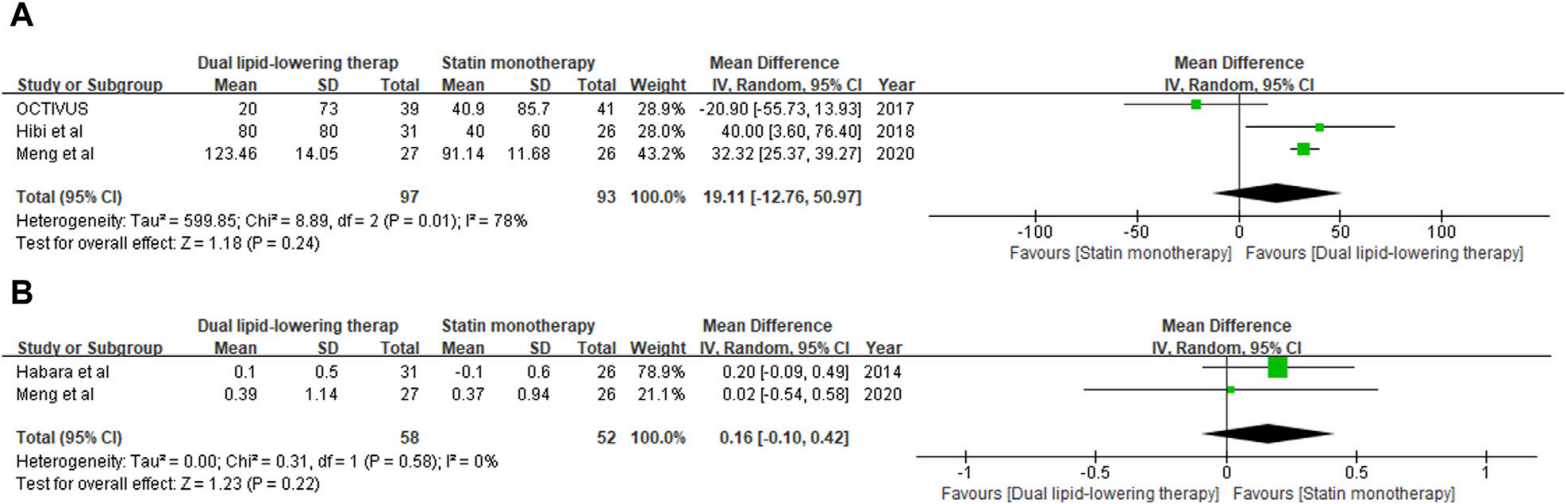
Forest plot of comparison showing the change in minimum fibrous cap thickness **(A)** and lumen area **(B)** assessed by OCT.

Plaque vulnerability was evaluated by minimum FCT derived from OCT examination. Data on the change from baseline in minimum FCT were available at the last follow-up for 180 individuals (97 in the dual-lipid-lowering therapy group and 93 in the statin monotherapy group) across three studies (Habara et al., 2014; Hougaard et al., 2020; Meng et al., 2020). With the pooled WMD of 19.11 (95% CI: 12.76–50.97), ezetimibe–statin combination therapy was revealed to be associated with a greater increase in minimum FCT than statin monotherapy. However, subsequent analysis revealed no statistical significance (*p* = 0.24). The outcomes are shown in Figure 4A.

Lumen stenosis of the target coronary artery plaque lesion was further verified by minimum LA through OCT examination. Data on the change from baseline in minimum LA were available at the last follow-up for 100 individuals (58 in the dual-lipid-lowering therapy group and 52 in the statin monotherapy group) across two studies (Habara et al., 2014; Meng et al., 2020). With the pooled WMD of 0.16 (95%: 0.10–0.42), ezetimibe–statin combination therapy exhibited a superior capability in restoring coronary lumen stenosis compared to statin monotherapy. However, subsequent analysis revealed that the difference was not statistically significant (*p* = 0.22). The outcomes are shown in Figure 4B.

### Trial sequential analysis (TSA)

**FIGURE 5:**
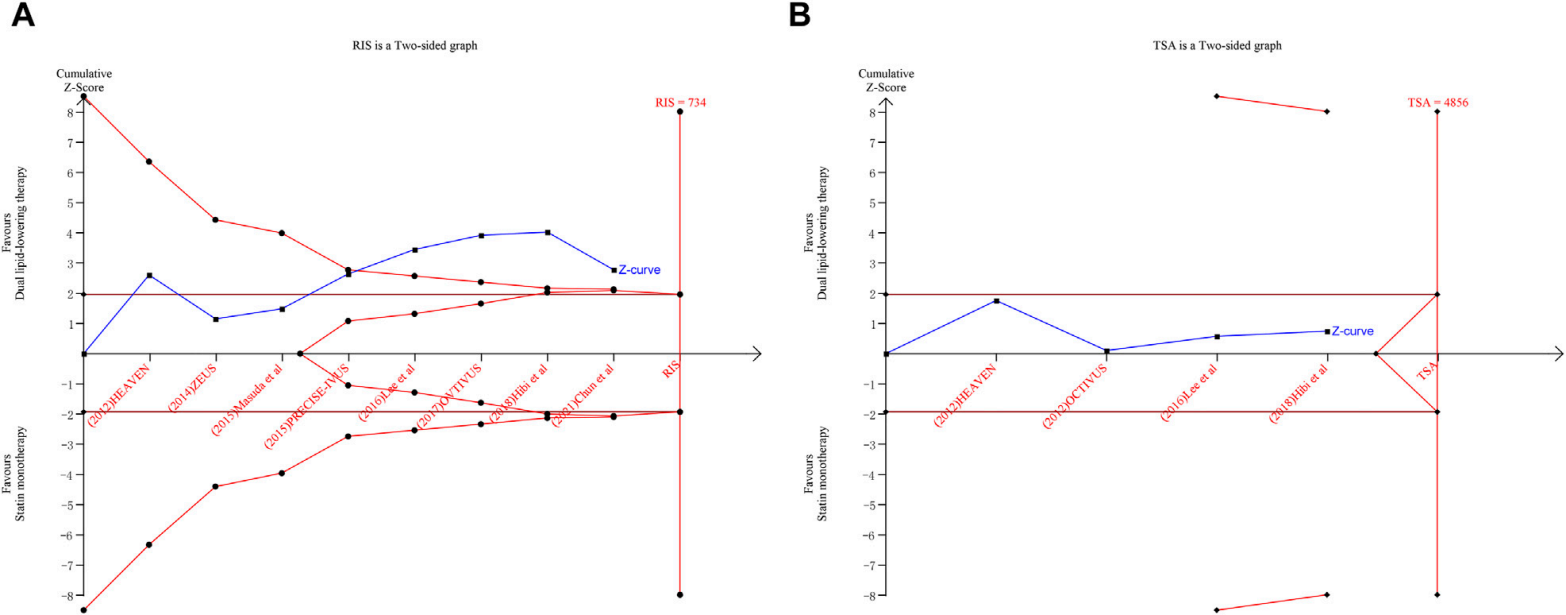
Trial sequential analysis for total atheroma volume **(A)** and minimum fibrous cap thickness **(B)**.

TSAs were performed for changes from baseline in TAV and minimum FCT. The Z-curve of TAV crossed both the traditional boundary values and the TSA boundary values. Although the accumulated information fell short of the required information size (RIS), the analysis demonstrated a statistically significant difference between ezetimibe–statin combination therapy and statin monotherapy in terms of efficacy on the regression of atherosclerotic plaque. The outcome is shown in Figure 5A.

However, the Z-curve of minimum FCT neither crosses the traditional boundary values nor the TSA boundary values. The accumulated information also fell short of the RIS. Although there was no statistically significant difference between the dual-lipid-lowering therapy and the statin monotherapy in terms of efficacy on plaque vulnerability, further studies directly evaluating plaque vulnerability with a large sample size are warranted. The outcomes are shown in Figure 5B.

### Publication bias

**FIGURE 6:**
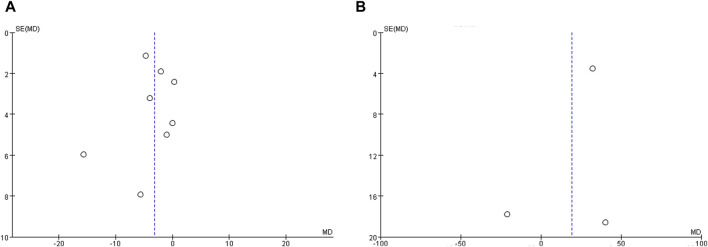
Funnel plot evaluating publication bias. **(A)** Funnel plots of studies reporting total atheroma volume as a measure of treatment effect, and **(B)** Funnel plots of studies reporting minimum fibrous cap thickness as a measure of treatment effect.

Publication bias statistics were assessed for studies reporting IVUS and OCT outcomes, respectively, by funnel plots. Figure 6 shows funnel plots of studies reporting the WMD of TAV and minimum FCT as indicators of the treatment effect. The plots demonstrate some mild asymmetry about the pooled effect, indicating the possibility of a few studies being omitted from inclusion and the existence of potential publication bias.

## Discussion

Two previous meta-analyses have assessed the effects of dual-lipid-lowering therapy with either ezetimibe or proprotein convertase subtilisin kexin type 9 inhibitor (PCSK9) in addition to a statin *versus* statin monotherapy on TAV derived from IVUS (Masson et al., 2020; Liang et al., 2022). Recognizing the potential clinical heterogeneity arising from a broad review scope, we decided to exclude studies evaluating the efficacy of evolocumab and alirocumab to ensure a more focused analysis, although the large patient population provided us with adequate statistical power to detect a treatment effect. Mirzaee et al. evaluated the influence of ezetimibe–statin combination therapy *versus* statin monotherapy on TAV through IVUS examination (Mirzaee et al., 2018). However, their quantitative meta-analysis included only six articles with a total of 583 subjects. Furthermore, all these studies aimed to evaluate the impact of such additive therapy on coronary plaque volume, overlooking changes in atherosclerotic plaque vulnerability and lumen stenosis of the coronary artery. Therefore, our study represents the first meta-analysis to evaluate the influence of ezetimibe in addition to statin schemes *versus* statin alone on coronary plaque modification, incorporating outcomes derived from intravascular IVUS and OCT, including coronary plaque burden, minimum FCT, and luminal stenosis. Through a systematic review of published evidence, 11 articles with a total of 926 individuals (460 in the dual-lipid-lowering therapy group and 466 in the statin monotherapy group) were included in the final meta-analysis.

IVUS is a catheter-based imaging modality that generates high penetrability images of the coronary wall, allowing the measurement of changes in the full thickness of the vessel wall. Total arthroma volume (TAV) derived from IVUS serves as a widely adopted and validated ultrasonic marker for quantification of the atherosclerosis burden. With the pooled WMD of −3.17 (95%CI: −5.42 to −0.92), it was revealed that ezetimibe–statin combination therapy led to significant regression in atherosclerotic plaque compared to statin monotherapy (*p* = 0.006). In other words, dual-lipid lowering regimens with ezetimibe added to statin might play a significant role in reversing plaque progression. On the other hand, the regression of atherosclerosis measured by IVUS has been a subrogated endpoint of clinical cardiovascular events (D'Ascenzo et al., 2013). This outcome helps explain the beneficial effects ezetimibe exerts on adverse cardiovascular events that are complementary to statin therapy. The findings of this meta-analysis align with the outcomes of the PRECISE-IVUS study, which demonstrated the favorable effect of ezetimibe in addition to statin therapy on the regression of coronary atheroma burden. Furthermore, subsequent TSA revealed that the Z-curve of TAV crosses both the traditional threshold and the TSA threshold, indicating that although the accumulated information does not reach the required information size, additional clinical trials are not required to obtain a positive conclusion.

Due to high spatial resolution, OCT is an imaging technology used to quantify coronary plaque microstructure *in vivo*. Minimum FCT serves as a crucial marker of plaque vulnerability and can be measured by OCT. A minimum FCT <75 μm is referred to as thin-capped fibroatheroma (TCFA), which exhibits the strongest correlation with clinical prognosis (Biccirè et al., 2023). Medications are known to stabilize atherosclerotic plaque with thickened fibrous layers. Our data demonstrated that ezetimibe–statin combination therapy led to a greater increase in minimum FCT than statin monotherapy. However, subsequent meta-analysis revealed no statistical significance (*p* = 0.24). Due to the limited sample size, TSA was employed to check whether the observed negative effect of ezetimibe-statin combination therapy on coronary plaque stabilization was true. TSA showed that further research with a larger sample size is still required. Furthermore, only three trials were eligible for statistical analysis, including one retrospective study (Meng et al., 2020) and two randomized controlled trials (Habara et al., 2014; Hougaard et al., 2020). Thus, our study remains inconclusive in terms of minimum FCT.

Small lumen area was previously reported to be a lesion-related risk factor for major adverse CV events (Stone et al., 2011). First, we took the LV derived from IVUS as effect size to evaluate the impact of ezetimibe added to statin therapy on lumen stenosis of the coronary artery. Compared with statin monotherapy, a subsequent meta-analysis revealed that no beneficial effect was associated with the ezetimibe–statin combination scheme [WMD = −0.52, 95%CI (−2.24 to 1.21), and *p* = 0.56]. To investigate whether ezetimibe–statin combination therapy has a similar effect to statin monotherapy, we took the minimum LA derived from OCT as a measurement of lumen stenosis of the coronary artery. With the pooled WMD of 0.16 (95%: 0.10–0.42), dual-lipid-lowing therapy exhibited a superior capability in restoring the coronary lumen compared to statin monotherapy. However, subsequent meta-analysis revealed no statistical significance either (*p* = 0.22). These outcomes align with the findings of the Simvastatin and Ezetimibe in Aortic Stenosis (SEAS) study, where no significant overall effect on the progression of aortic stenosis, as observed on echocardiography, was demonstrated with ezetimibe–simvastatin combination therapy over a minimum period of 4 years (Rossebø et al., 2008).

The findings of this study should be interpreted with recognition of the inherent limitations. First, there is clinical heterogeneity across eligible studies that cannot be completely resolved by statistical analysis despite the use of a random-effects model. Specifically, study designers selected volunteers primarily based on angiographic lumen stenosis and baseline LDL-C concentration with little attention to medical history. A sub-analysis of the PRECISE-IVUS trial concluded that the atorvastatin/ezetimibe combination was associated with a significantly stronger reduction in atheroma volume in patients with statin pretreatment (Tsujita et al., 2016a). Thus, the treatment response to the ezetimibe–statin dual-lipid-lowering therapy may vary based on the patient sample (e.g., statin-naïve *versus* statin-pretreated patients). Furthermore, the type and dosage of statin varied greatly across studies. Unfortunately, the interaction of these clinical variances with the treatment effect of dual-lipid-lowering therapy cannot be analyzed with aggregate patient data, and ideally, data from individual patients should be analyzed (Lyman and Kuderer, 2005). Second, the estimated effect in this meta-analysis is constrained by study design. Although most of the included studies were RCTs, some of them were open-label trials that did not blind participants. Additionally, one non-randomized trial (Nakajima et al., 2014) and one retrospective study (Meng et al., 2020), both more susceptible to various biases, were included in the final meta-analysis. Moreover, confounding factors that could be balanced by randomization in RCTs often complicate the observation of intervention effects. It is, therefore, not surprising that almost all of these studies were assessed to be at a relatively high risk of bias (Figure 2). Third, the overall impact of dual-lipid-lowering therapy with ezetimibe in addition to statin might be underestimated. Specially, the sample size of eligible studies was relatively small, and the follow-up duration of treatment was relatively short, ranging from 3 to 12 months, with no studies having a long follow-up. The relationship between treatment duration and plaque regression remains unknown. Thus, it is necessary for clinicians to interpret our findings carefully.

In conclusion, this meta-analysis demonstrated that dual-lipid-lowering therapy, involving the addition of ezetimibe to statin, offers incremental benefits in reducing coronary plaque burden compared to statin monotherapy. However, no statistically significant difference was observed in lumen volume or minimum lumen area. Furthermore, ezetimibe–statin combination therapy did not show a significant impact on minimum FCT.

## Impact on daily practice

The current meta-analysis confirms the plaque regression abilities of dual-lipid lowering treatment with ezetimibe and statins. However, the addition of ezetimibe to statin does not help with plaque stabilization and diameter stenosis. Nevertheless, further prospective randomized controlled studies with larger sample sizes are still warranted.

## Data Availability

The original contributions presented in the study are included in the article/Supplementary Material, further inquiries can be directed to the corresponding author.
